# Extracellular DNA in natural environments: features, relevance and applications

**DOI:** 10.1007/s00253-018-9120-4

**Published:** 2018-06-01

**Authors:** Magdalena Nagler, Heribert Insam, Giacomo Pietramellara, Judith Ascher-Jenull

**Affiliations:** 1 Universität Innsbruck, Institute of Microbiology, Technikerstr. 25d, 6020 Innsbruck, Austria; 2 Dipartimento di Scienze delle Produzioni Agroalimentari e dell’Ambiente, Università degli Studi di Firenze, Piazzale delle Cascine 18, 50144 Florence, Italy

**Keywords:** Extracellular DNA, Environment, Biofilm, Soil, Plant, Microbial activity

## Abstract

Extracellular DNA (exDNA) is abundant in many habitats, including soil, sediments, oceans and freshwater as well as the intercellular milieu of metazoa. For a long time, its origin has been assumed to be mainly lysed cells. Nowadays, research is collecting evidence that exDNA is often secreted actively and is used to perform a number of tasks, thereby offering an attractive target or tool for biotechnological, medical, environmental and general microbiological applications. The present review gives an overview on the main research areas dealing with exDNA, depicts its inherent origins and functions and deduces the potential of existing and emerging exDNA-based applications. Furthermore, it provides an overview on existing extraction methods and indicates common pitfalls that should be avoided whilst working with exDNA.

## Introduction

In contrast to intracellular DNA (iDNA), which is the DNA located within cell membranes, extracellular DNA (exDNA) represents the DNA located outside thereof. Such DNA can be found in any kind of environmental samples. One of the best definitions including information about its origin was given by Pietramellara et al. (2009), stating that exDNA is “originating from intracellular DNA by active or passive extrusion mechanisms or by cell lysis”.

Dealing with environmental DNA, several abbreviations are used to refer to similar or different items. Whilst a common acronym for environmental DNA is eDNA, a number of authors used this acronym for extracellular DNA, too. Additionally, the terms exDNA or cfDNA (cell-free DNA) were introduced to refer to extracellular DNA in order to prevent confusion with environmental DNA. In this review, we give the preference to the acronym exDNA. Marine biologists often differentiate between aqueous-extractable “soluble DNA” (sDNA) and “non-soluble DNA” (nsDNA); both fractions are roughly representing exDNA and iDNA, respectively (Lever et al. 2015). The acronyms esDNA, aDNA and cirDNA stand for extracellular self-DNA, ancient DNA and circulating DNA, respectively, and will be addressed in the chapters “soil”, “marine and lake ecosystems” and “human body”, respectively.

When it has become known to be common in the environment in the early 1950s, exDNA was studied in the context of horizontal gene transfer (HGT) (Avery et al. 1944; Freeman 1951) and the ability of microorganisms to achieve antibiotic resistance through transformation by foreign (extracellular plasmid) DNA (Akiba et al. 1960; Romanowski et al. 1993). During the 80s and 90s of the past century, exDNA was studied in terms of its persistence in soil, i.e. protection against nuclease degradation due to binding to various soil components (Ogram et al. 1987; Paget et al. 1992; Vettori et al. 1996), and its degradation rates in estuarine and marine environments (Paul et al. 1987).

Frostegård et al. (1999) evaluated DNA extraction efficiencies of several protocols and addressed the issue of extracting extracellular and intracellular soil DNA simultaneously. By then, exDNA was found to be omnipresent, and with this awareness, a variety of research foci on different natural environments emerged:

- The persistence and ecological relevance of exDNA in soil (reviewed by Levy-Booth et al. 2007; Pietramellara et al. 2009);
- The persistence, function and turnover of exDNA in marine and aquatic ecosystems (reviewed by Torti et al. 2015);
- The occurrence, relevance of exDNA and possible exDNA-derived therapies in the human body (reviewed by Aucamp et al. 2016; Cooper et al. 2013; Thierry et al. 2016);
- The importance and the functions of exDNA in the formation of biofilms of pathogenic and environmental microorganisms (reviewed by Montanaro et al. 2011 and Wnorowska et al. 2015 (exDNA), Hobley et al. 2015 (biofilms in general), Wolska et al. 2016 (genetic control), Payne and Boles 2016 (matrix interactions and resulting implications) and Azeredo et al. 2017 (methods)).

Extracellular DNA has also been investigated within dead wood (Gómez-Brandón et al. 2017a), cattle rumen and manure (Chroňáková et al. 2013; Fliegerová et al. 2014; Nagler et al. 2018), aerobic and anammox granules (Cheng et al. 2011; Xiong and Liu 2012; Dominiak et al. 2011) and human epithelial cells used in forensics (Wang et al. 2017). In addition, exDNA was found to act as a trap for infectious organisms in mammalians (reviewed by Ciesluk et al. 2017) and during root tip growth of plants (Hawes et al. 2012; Pietramellara et al. 2013). Finally, exDNA is assumed to act as a species-specific growth inhibitor all over the tree of life (Mazzoleni et al. 2015b; esDNA).

**Fig. 1:**
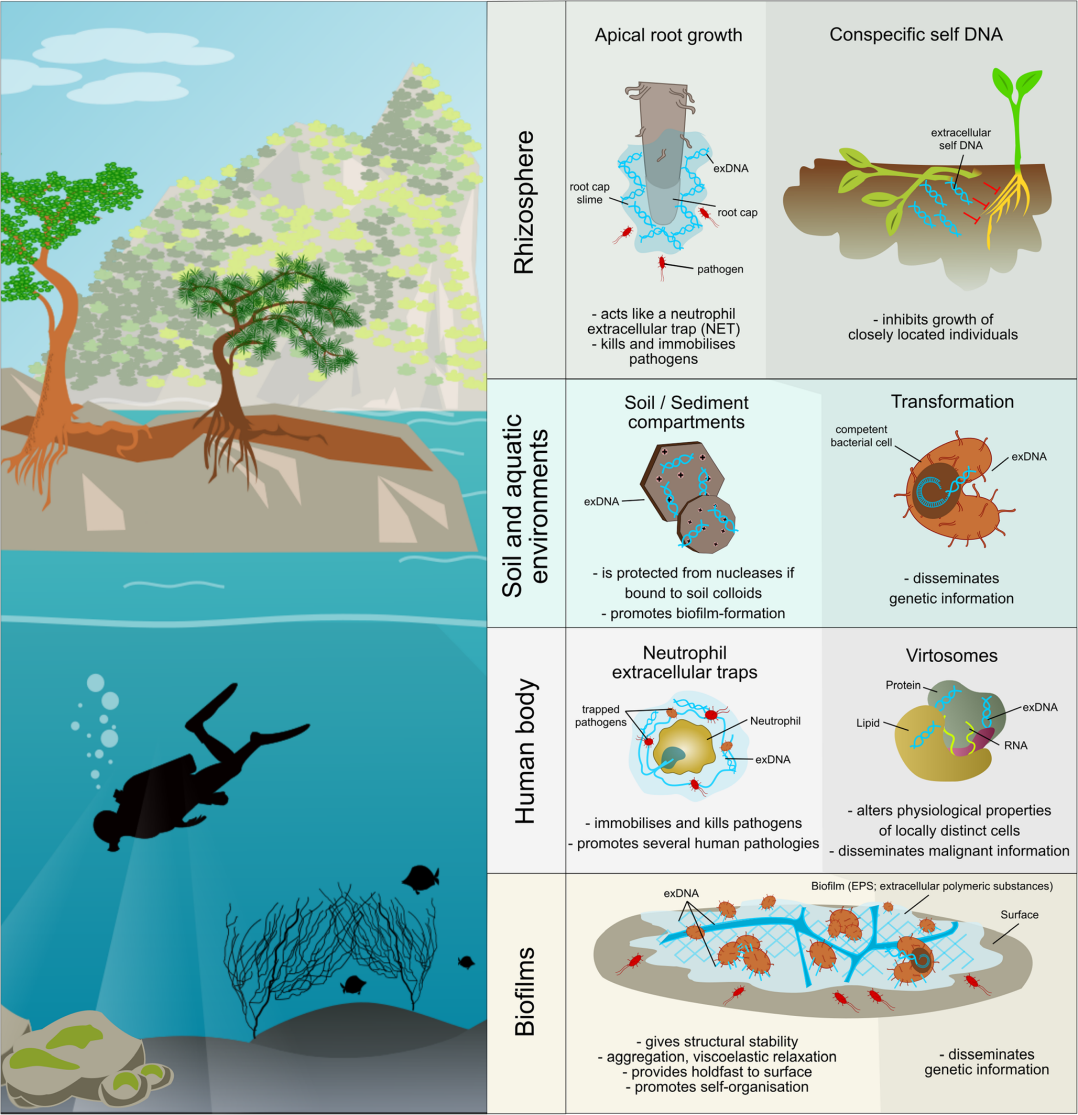
Main functions of extracellular DNA (exDNA) in different natural environments. Darker shaded areas represent functions deriving from the informational character of exDNA, whilst lighter areas comprise functions owed to the “sticky” character of exDNA

Whilst most of recently published reviews regarding exDNA focus on a single specific environment, the present review aims to summarise the main features, functions and pertinences of exDNA in all so far investigated natural environments (Fig. 1). In doing so, we also intend to depict existing as well as emerging exDNA-based applications. Furthermore, we give a short overview on existing extraction methods and indicate common pitfalls that should be avoided whilst working with exDNA.

## Soil

In soils, exDNA is omnipresent and has first been studied with regard to its adsorption to sand, clay and other soil colloids (Fig. 1) (e.g. Lorenz and Wackernagel 1987; Paget et al. 1992; Pedreira-Segade et al. 2018). Once bound to these particles, exDNA is partly physically protected from degradation, allowing persistence for years (Agnelli et al. 2007; Nielsen et al. 2007). The actual persistence of exDNA depends on a number of factors such as its composition, methylation or conformation and the prevailing environmental conditions. In that context, rapid desiccation, low temperatures, high salt concentrations, low pH and a high content of expandable clay minerals have all been found to slow down exDNA degradation (Crecchio et al. 2005; Pietramellara et al. 2009). An attempt to estimate the age of soil exDNA by radiocarbon dating suggested a survival time ranging from 21,000 years (^14^C age) to 900,000 years (mean residence time), even though it was suggested to treat these results with care, as a contamination (e.g. with fossil carbon) could not be totally excluded (Agnelli et al. 2007). Despite its binding to various minerals, exDNA still preserves its ability to transform competent microbial cells in the soil (Fig. 1) (Morrissey et al. 2015; Romanowski et al. 1993; Thomas and Nielsen 2005). Whilst some studies suggested that HGT frequencies in soil are low (Nielsen et al. 1998; Pietramellara et al. 2007; Pietramellara et al. 2006; Thomas and Nielsen 2005), some hypothesised that the actual transformation rates are underestimated due to the high number of unculturable microorganisms (Pietramellara et al. 2009). However, the long persistence of DNA in soil brings about an increased presence of antibiotic resistance genes that might be passed from cell to cell (Poté et al. 2003), with both ecological and evolutionary implications. The quality of exDNA is depending on its state of degradation, fragment sizes ranging from 80 to more than 20,000 bp, as shown by standard agarose gel electrophoresis (Ascher et al. 2009b). The integrity of large fragments of exDNA was shown by the successful amplification of a 1700-bp portion, almost the complete fungal 18S gene (Ascher et al. 2009b). A large percentage of exDNA in soil was found to be double stranded, being detectable with methods specifically binding to double-stranded DNA (intercalation dyes, e.g. PicoGreen) (Agnelli et al. 2004; Ascher et al. 2009b).

After active or passive excretion or release from lysed cells (i.e. after cell death/necrosis or virus attack), exDNA can be diffused in the soil through various mechanisms. Vertically, the movement was found to be either directed towards the groundwater through leaching or towards the soil surface through advection in water capillaries; horizontally, movement follows the soil water flow direction (Agnelli et al. 2004; Ascher et al. 2009a; Ceccherini et al. 2007; Poté et al. 2003). In both directions, exDNA may reach areas with little nutrient content. Accounting for over 10% of the extractable P in soil and containing essential elements such as N and C, exDNA may act as a nutrient and energy source especially in soils with low nutrient input (reviewed by Levy-Booth et al. 2007; Nielsen et al. 2007). After a breakdown by extracellular and cell-associated nucleases (DNases), smaller exDNA molecules are taken up by microbial cells, where they either serve as building blocks for newly synthetized nucleic acids or are further broken down to essential nutrients (Morrissey et al. 2015).

Just like in other environments, soil exDNA plays a crucial role in the formation of biofilms, exhibiting mainly structural functions as discussed below and serves as an information pool for HGT. Similarly, soil particles and organisms such as microalgae and microorganisms are known to form biological soil crusts particularly in the topsoil of arid soils, where the production of extracellular polymeric substances (EPS) including exDNA leads to an increased water retention (e.g. Adessi et al. 2018). Such soil-microbe systems are thought to be self-organised in a way that microbes shape the state of oxygen supply through their activity (respiration), causing a shift between oxygen supply and high potential activity on the one hand and protection from desiccation and predation in a low-potential activity regime on the other (Young and Crawford 2004). Supporting the formation of pores and aggregates according to its structural properties, exDNA could possibly contribute to this self-organisation.

Bearing additional taxonomic and phylogenetic information with regard to iDNA, exDNA has therefore been used to compare information about microbial communities deriving from both fractions of the total soil DNA pool (Agnelli et al. 2004; Ascher et al. 2009b; Ceccherini et al. 2009; Chroňáková et al. 2013; Gómez-Brandón et al. 2017b). These studies revealed that some sequences found in the exDNA fraction are not found in the iDNA fraction of the total DNA pool and suggest that they are ancient or so-called relic DNA. Such DNA, potentially persisting in soil for long time spans, reflects the historical biodiversity of the investigated environment and can give important information about past climatic conditions (see the “Applications” section). A study conducted by Carini et al. (2016) actually showed that the exDNA inflated the observed prokaryotic and fungal richness by up to 55% if compared to iDNA only. Following these findings, it was argued that the quantitatively relevant presence of exDNA might also cause an underestimation of the actual temporal and spatial variability of soil microbial communities (Fierer 2017). This may put a new perspective on the concept of “everything is everywhere, but the environment selects”, stating that most species are present at least in low abundances in all soils and will thrive as soon as the environmental conditions allow for (Baas Becking 1931; Fenchel and Finlay 2004; Nagler et al. 2016). For any assumptions concerning diversity and microbial species abundance, it is thus indispensable to distinguish between environmental DNA (eDNA) and exDNA on the one hand, and the extracellular (exDNA) and intracellular fraction (iDNA) of the total DNA pool on the other (reviewed by Taberlet et al. 2012a) (see the “Applications” section).

In an investigation on litter autotoxicity, the role of extracellular self-DNA (esDNA) has first been addressed by Mazzoleni et al. (2015a), who found that the growth not only of plants but also of soil animals and microorganisms was inhibited when conspecific exDNA was added to the growth substrate (Mazzoleni et al. 2015a, b). This effect was found to be very specific and applied only for conspecific but not for other heterologous exDNA. The authors hypothesised that this inhibition effect represents a mechanism of maintaining diversity. In an attempt to interpret these far-reaching findings, Veresoglou et al. (2015) discussed that esDNA in soil could function as a conspecific stress-signalling molecule rather than an inhibitory substrate. Similarly, Duran-Flores and Heil (2015) argued that esDNA could belong to the group of damage-associated molecular patterns (DAMP) that cause the local development of resistance-related responses by the affected plant. All these findings, however, are rather preliminary and require additional research to adequately interpret and describe the underlying mechanisms.

Finally, the role of exDNA in soil is also linked to plant physiology. The presence of exDNA in the growth medium of plants enhances the growth of lateral roots and root hairs and the effect is linked to an altered expression of specific peptide hormone genes that are controlling root morphology (Paungfoo-Lonhienne et al. 2010). In that context, exDNA has the function of a signalling compound. In the context of root growth itself, its role is different. Wen et al. (2009) reported that exDNA is a component of the root cap slime known to be involved in the increased resistance of growing root caps against soil-borne pathogens, and that exDNA degradation resulted in a loss thereof (Wen et al. 2009). Later on, several studies suggested that exDNA actively exported from the root tip may function similar to the exDNA secreted in human neutrophil extracellular traps (NETs) and traps pathogenic microorganisms in close proximity to the root tips (reviewed by Hawes et al. 2011): once released by active secretion (Wen et al. 2017), the exDNA attracts and immobilises pathogens as well as soil contaminants in a host-microbe specific manner (Hawes et al. 2012; Hawes et al. 2016; Pietramellara et al. 2013).

Not strictly soil but still closely related, antimicrobial resistance might emerge with increased frequency in livestock waste management structures. Zhang et al. (2013) found that several antimicrobial resistance genes were present in the exDNA and iDNA pool of such environments and that HGT is a potential mechanism for the spread of antimicrobial resistance. Investigating rumen-borne microbial communities, considerable differences between exDNA and iDNA bacterial profiles have been found (Fliegerová et al. 2014), suggesting differing lysis and/or DNA secretion of the microorganisms.

## Marine and aquatic ecosystems

In the marine environment, exDNA is present throughout, from the estuarine to the anoxic deep sea. Its origin, dynamics and implications have been reviewed by Torti et al. (2015). It is estimated that around 90% of the total DNA pool in the ocean occur as exDNA (Dell'Anno and Danovaro 2005), which accounts for a global 0.45 Gt of DNA in the uppermost 10 cm of sea water, where amounts of exDNA are three orders of magnitudes lower than in sediments (Torti et al. 2015). Marine exDNA is either autochthonous or allochthonous, passively or actively released from decaying, virus-attacked or growing (micro)organisms. If the exDNA is released in the water column, it sediments only if complexed with particles heavy enough to sink to the sea floor (Herndl and Reinthaler 2013). However, once released, the fate of exDNA includes natural transformation, degradation through ubiquitous DNases and subsequent incorporation by microbial cells, long-term preservation and abiotic decay (Fig. 1). As for long-term preservation, binding of exDNA in marine sediments is similar to that of soil; the interaction is electrostatic and requires the presence of inorganic cations to bind the negatively charged inorganic and organic sediment surfaces with the phosphate groups of DNA (Fig. 1) (Lorenz and Wackernagel 1987). Furthermore, exDNA is preserved after contact with brines of deep anoxic hypersaline lakes (Borin et al. 2008), where non-adapted bacteria might lyse with a higher frequency due to osmotic stress, giving rise to an environment favouring high rates of HGT.

Next to exDNA in the water column and in the sediments, exDNA can also be located in the extracellular polymeric substance (EPS) of marine biofilms, as reviewed by Decho and Gutierrez (2017). EPS form a major component of the total pool of dissolved organic carbon in the ocean, but the role of exDNA in this specific environment has not been investigated so far.

Regarding lake and other freshwater environments, exDNA-related studies are very scarce. A study reporting about ferruginous sediments in a tropical lake in Indonesia used the exDNA bound to the sediment to study the microbial consortium and detected exDNA in decreasing amounts from the lake ground to 30-cm sediment depth as well as differences in the taxonomic composition between exDNA and iDNA (Vuillemin et al. 2016). Another study focussed on the persistence of antimicrobial resistance genes in the exDNA pool of a river sediment and reported that resistance genes often incorporated into plasmid DNA exhibit a longer persistence than chromosomically encoded 16S rRNA genes, suggesting that exDNA represents a major reservoir for antibiotic resistance information (Mao et al. 2014). In the Arctic sea ice, exDNA has been found in concentrations higher than those reported from any marine environment and it was hypothesised that sea ice may be a hotspot for HGT in the marine environment (Collins and Deming 2011).

## Biofilms

One of the best-studied environments housing exDNA are biofilms, the focus lying particularly on those formed by clinically relevant microorganisms such as *Staphylococcus* spp., *Streptococcus* spp., *Candida* spp., *Pseudomonas aeruginosa* and mixed oral biofilms. Other biofilms formed by environmental microorganisms, plant pathogens (Sena-Velez et al. 2016), or in the activated sludge during wastewater treatment have been studied to a lesser extent (e.g. Dominiak et al. 2011).

The presence of DNA in the EPS and its responsibility for the stickiness of the by then so called “slime” or “mats” was discovered as early as in 1955 for some halophilic bacteria (Smithies and Gibbons 1955) and several years later with a focus on human pathogens for *Pseudomonas aeruginosa* (Murakawa 1973). Beginning in 1996, exDNA was increasingly noted in the EPS matrix of activated sludge and in pure cultures of *Pseudomonas putida* (reviewed by Flemming and Wingender 2010). The origin of this DNA has long thought to be lysed cells. Later, it was found that the exDNA is present in species-specific amounts in different single- and multiple-species biofilms (Steinberger and Holden 2005) and that it is organised in clear patterns, forming grid-like structures or filamentous networks (Fig. 1) (Allesen-Holm et al. 2006; Böckelmann et al. 2006; Flemming et al. 2007). As a consequence, exDNA has been described as a structural component of the extracellular matrix, being essential especially during biofilm formation (Conover et al. 2011; Kawarai et al. 2016; Martins et al. 2010; Novotny et al. 2013; Nur et al. 2013; Seper et al. 2011; Whitchurch et al. 2002; Zhao et al. 2013) (reviewed by Flemming et al. 2016; Montanaro et al. 2011) and thus being actively secreted by the biofilm-producing microorganisms (Barnes et al. 2012; Kilic et al. 2017; Liao et al. 2014; Rose and Bermudez 2016; Zafra et al. 2012). A genome-wide screening for genes involved in exDNA release during biofilm formation by *S. aureus* was recently done (DeFrancesco et al. 2017).

In biofilms of mixed bacterial consortia such as granular activated sludge, differences in the composition of exDNA vs. iDNA were detected applying a fingerprinting approach (Cheng et al. 2011) and indicating a species-specific DNA release originating mostly from active secretion (Dominiak et al. 2011). Moreover, microbial aggregation during aerobic granulation and consequently biomass density and size are positively affected by increased exDNA amounts (Xiong and Liu 2012). In oral biofilms, the exDNA consists not only of microbial but also of host-DNA but exhibits similar functions than in other biofilms (reviewed by Jakubovics and Burgess 2015; Schlafer et al. 2017).

Focusing on the role of exDNA in biofilms, several studies (Doroshenko et al. 2014; Hathroubi et al. 2015; Schilcher et al. 2016) found increased exDNA concentrations after exposure to low concentrations of antibiotics and vice versa, a higher antimicrobial resistance with higher amounts of exDNA (Johnson et al. 2013; Lewenza 2013), suggesting a protective function. Through its negative charge, exDNA acts as a chelator of cationic antimicrobials (Mulcahy et al. 2008) but can also act as a protection system against aminoglycosides (Chiang et al. 2013). The main protective power against antimicrobials or predation, however, is owed to the exDNA’s function to structurally stabilise biofilms and thereby increase antimicrobial resistance (see the “Applications” section). exDNA has also been shown to attract and bind with positively charged amyloids in various biofilms, thereby accumulating peptides and causing a polymerisation of the matrix and stimulating autoimmunity (reviewed by Payne and Boles 2016; Randrianjatovo-Gbalou et al. 2017; Schwartz et al. 2016). An interaction with polysaccharides was found in *P. aeruginosa* and *S. mutans* biofilms, where both components form a web of fibres and function as a skeleton allowing bacteria to adhere and grow (Payne and Boles 2016; Pedraza et al. 2017).

The role of exDNA as a source of genetic information in the context of HGT within the biofilm has been addressed in several studies (e.g. Merod and Wuertz 2014; Wang et al. 2002) and was found to occur frequently, as biofilms are hotspots, i.e. offer ideal conditions for HGT including high cell density, increased genetic competence and an accumulation of exDNA. Conjugation has been shown to be up to 700-fold more efficient in biofilms compared to planktonic bacterial cells (Flemming et al. 2016), further promoting antimicrobial resistance in biofilms. Moreover, several other functions of exDNA in biofilms have been described. In most biofilms, exDNA is needed throughout the biofilm development (Brockson et al. 2014) but especially for the initial adhesion and aggregation of bacteria on surfaces (Das et al. 2010; Das et al. 2011; Jermy 2010; Tang et al. 2013). In *Caulobacter crescentus* biofilms, however, exDNA binds to the holdfast of swarmer cells, promotes their dispersal to places with less present exDNA and thereby prevents biofilm maturation (Berne et al. 2010; Kirkpatrick and Viollier 2010). Furthermore, it has been suggested that self-organisation of cells in actively expanding biofilms of *P. aeruginosa* occurs directly on the exDNA filaments (Böckelmann et al. 2006) or through the construction of a network of furrows supported by exDNA molecules (Gloag et al. 2013). During mechanical stress of a biofilm, exDNA was found to exhibit a distinguishable role in controlling the viscoelastic relaxation of the biofilm (Peterson et al. 2013). In addition, Sapaar et al. (2014) suggested that exDNA may induce the morphological change from yeast to hyphal growth in *C. albicans* biofilms, but without providing any explanation about the possible underlying mechanisms.

## Human body

Next to the exDNA secreted by clinically relevant microorganisms forming biofilms on or inside the human body (covered in the section *biofilm*), exDNA of predominantly endogenous origin can be found in the extracellular milieu of the human body including blood, lymph, bile, milk, urine, saliva, mucous suspension, spinal and amniotic fluid. Beginning in the 1960s, exDNA was discovered in the plasma and serum of patients with a variety of diseases, including rheumatoid arthritis, pancreatitis, inflammatory bowel disease, hepatitis and oesophagitis. By the 1970s, it was shown to be double-stranded and of a similar size range as in soil (i.e. from 180 to 10,000 bp) (van der Vaart and Pretorius 2008). With the development of more sensitive assays, it was found to be also present in healthy subjects, albeit to a lesser extent (Anker et al. 1999). It has been proposed that this type of circulating exDNA (cirDNA, cell-free cfDNA or plasma DNA) is released by apoptosis and necrosis, by bacteria and viruses, and via active release from highly proliferating cells (reviewed by Thierry et al. 2016). Anker et al. (1976) obtained evidence that human lymphocytes can release complexes containing DNA or produce enzymes that are capable of synthesising DNA extracellularly. If originating, however, from such an active cellular release mechanism, exDNA is often bound to other plasma constituents such as RNA, lipids and proteins, being in that case called virtosomes (Fig. 1). As part of virtosomes, exDNA shows the ability to migrate to different parts of the body, enter target cells and alter their physiological properties such as the immune response, by sharing antigenic information (Anker et al. 1984; Aucamp et al. 2016; Skog et al. 2008). Peters and Pretorius (2012) highlighted that this active release and uptake of nucleic acids is a characteristic of all organisms and cell types, and that in contrast to the neo-Darwinian dogma, physical and behavioural traits can be inherited through this cycling. This is because there has been found evidence that not only somatic but also germ cells might be subject to genetic and epigenetic modifications via exDNA (intensively reviewed and discussed by Aucamp et al. 2016). In this context, it has been hypothesised that the exDNA in human blood vessels might derive to a large extend from metabolic DNA, which is—as opposite to the stable genetic DNA—a specially synthesised low-molecular-weight fraction of DNA involved in the regulation and performance of RNA production and other cellular functions. Deriving from such a de novo synthesis in cells (van der Vaart and Pretorius 2008), exDNA differs from the DNA in the nucleus containing single- and double-strain breaks and accumulations in GC-rich regions (Veiko et al. 2008).

Another field of studies regarding exDNA in the human body is the immune system, where neutrophils secrete exDNA together with actin, histone, peroxidases and proteins, thereby forming a neutrophil extracellular trap (NET), a sticky matrix around the cell (Fig. 1) (Brinkmann et al. 2004). These NETs are part of the immune system and are formed as a response to defence-pathway-inducing signals. Pathogens can be chemotactically attracted by the NETs and are then immobilised and potentially killed by the antimicrobial components of the trap (Halverson et al. 2015; Hawes et al. 2015). Hawes et al. (2015) proposed that NETs gain most of their bactericidal character through the removal of surface-stabilising bacterial cations by the DNA phosphodiester backbone, resulting in bacterial lysis. Recent studies, however, revealed that an overproduction of NETs followed by an accumulation of exDNA contributes to the pathogenesis of some diseases. Breast cancer cells can induce neutrophils to produce NETs without infection (Park et al. 2016), thereby exploiting the host cells in order to promote metastases. Furthermore, NETs can cause the aggregation and implantation of cancer cells due to its *sticky* character (Hawes et al. 2015). In this context, the genometastasis hypothesis was formulated, stating that exDNA derived from tumour cells just like virtosomes can enter healthy cells and lead to the formation of metastases as reviewed by García-Olmo et al. (2012) and discussed by Thierry et al. (2016). A variety of other pathologies have recently been linked to exDNA, including the chronic airway disease, where NETs accumulate in the airways leading to an activation of the innate immune system and impairing the patients’ state of health (Wright et al. 2016). Similarly, in patients suffering from dry eye disease, the production and degradation of exDNA is altered, allowing exDNA and NETs to accumulate in the tear film and resulting in an inflammation (Sonawane et al. 2012; Tibrewal et al. 2013).

On the one hand, the functional role of exDNA inside the human body and especially the blood vessels is to serve as an *intercellular messenger* in the shape of virtosomes (Gahan and Stroun 2010), spreading the immunological information about pathogenic invaders but also supporting the dissemination of malignant information causing oncogenesis, cell invasion, metastasis and the development of resistance against radiotherapy and chemotherapy (Aucamp et al. 2016). On the other hand, exDNA has been shown to act as a trap for invading pathogens in the shape of NETs, being in that way a part of the innate immune system and combatting an infection. The same benign NETs can cause, however, pathophysiological effects in cancer, autoimmune pathologies, sepsis, thrombotic illness and in the inflammatory response through different mechanisms, as highlighted above (Ciesluk et al. 2017; Cooper et al. 2013; Park et al. 2016).

## Methodological considerations with exDNA extraction

Extraction methods targeting exDNA vary amongst environmental matrices. In the soil, exDNA can strongly be bound to soil colloids like clay minerals or humic acids, resulting in a co-extraction of organic and inorganic soil compounds interfering with downstream analyses. To overcome these problems and prevent a lysis of intact cells, exDNA is desorbed from soil particles via slightly alkaline solutions or phosphate buffers and yielded in the supernatant after centrifugation, avoiding the use of cell-lysing reagents and optionally including DNase inhibitors (e.g. Agnelli et al. 2007; Ascher et al. 2009b; Ceccherini et al. 2009; Ogram et al. 1987; Taberlet et al. 2012b). Applying such a sequential extraction, next to providing exDNA, increases the total amount of not only extractable soil DNA but also that of iDNA (e.g. Ascher et al. 2009b; Nagler et al. 2018; Wagner et al. 2008).

Analogously, a discrimination between sDNA and nsDNA is proposed in marine sediment studies, applying a washing in alkaline phosphate buffers followed by centrifugation prior to standard DNA extraction (Alawi et al. 2014; Lever et al. 2015). During sampling of exDNA from water samples, a filtration through filters retaining the exDNA is required and it was found that the binding of exDNA is significantly differing with filter material, pore size and several water quality parameters such as pH or total suspended solids (Liang and Keeley 2013).

In biofilm research, exDNA extraction without contamination of genomic DNA was found to work best with enzymatic treatment methods yielding more exDNA than a simple centrifugation (Wu and Xi 2009). In cancer research, a discrimination between weakly bound and tightly bound exDNA is made, and accordingly, a first step to remove weakly bound exDNA is applied using 5 mM EDTA and a second step using trypsin to remove exDNA tightly bound to cell surfaces is suggested (Laktionov et al. 2004).

In general, independent of the environmental matrix, any harsh step (physico-chemical) has to be avoided during the extraction procedure, so as to avoid potential cell lysis.

## Applications

### exDNA as source of specific genetic information

One of the most immanent features of exDNA is the additional phylogenetic information with respect to iDNA. Therefore, exDNA can be used to improve the accuracy of assessing the soil microbial community composition (Pietramellara et al. 2009), e.g. via comparative genetic fingerprinting of the extracellular and intracellular fraction of the total DNA pool (Agnelli et al. 2004; Ascher et al. 2009b; Chroňáková et al. 2013) or via quantitative PCR (Gómez-Brandón et al. 2017a, b).

### exDNA as a proxy of microbial activity (microbial turnover)

Another feature is the origin of exDNA in various environments, which was expected to be mainly lysed (dead) cells (Levy-Booth et al. 2007), whilst iDNA is attributed to intact (alive and potentially alive) cells. Consequently, a ratio of both DNA fractions (exDNA:iDNA) might provide a reliable approximate measure for microbial activity in soils and other environments (Gómez-Brandón et al. 2017a, b; Nagler et al. 2018). Surprisingly, the activity of different microbes was found to not correlate perfectly with the ratio of exDNA:iDNA but could best be tracked measuring exDNA amounts without relation to iDNA (Nagler et al. 2018). These results suggested that exDNA is released by microorganisms proportional to their activity. Similarly, Dlott et al. (2015) found a unexpected low rRNA:rDNA ratio when trying to establish a method to measure individual microbial activity and these ratios were due to high amounts of amplifiable exDNA. Both results suggest that the exDNA fraction, which is suitable in its quality for a qPCR or other downstream molecular methods, seems to derive to a large part from actively released DNA and might thus reflect microbial activity, whilst the exDNA deriving from lysed cells is not yielded using these methods. These results should be considered when applying methods such as the viability PCR (Emerson et al. 2017; Nocker et al. 2006; Wagner et al. 2008) or a treatment with DNase I/proteinase K (Villarreal et al. 2013). These methods are based on the assumption that exDNA mainly derives from dead cells. Consequently, iDNA and total DNA are measured by a degradation of the exDNA in one of two parallel samples to give a live/dead ratio. In fact, exDNA may not only have derived from recently lysed and active cells but may also be relic DNA that has persisted outside of intact cell membranes for decades and centuries, especially when bound to inorganic particles such as soil colloids. Thus, an activity tracking using exDNA should be further investigated considering this ancient exDNA probably being present at a low but stable rate in a variety of environments.

### exDNA as specific target matrix for (prokaryotic and eukaryotic) biodiversity survey studies

Within the field of environmental DNA research (Thomsen and Willerslev 2015), a recent approach focused on the extracellular fraction of environmental DNA and aimed to study the soil biodiversity at large scale (landscape scale; e.g. vegetation map) from large and thus representative sample volumes by applying a metabarcoding approach (e.g. Orwin et al. 2018; Taberlet et al. 2012b). However, quantitative as well as qualitative conclusions should be interpreted with caution, as the results might be influenced by actively released and ancient exDNA.

### exDNA as tool for evolution research

In the field of marine biology, the identification and enumeration of microscopic remains in sediments such as fossilised protists can be supported studying ancient exDNA (aDNA), being reported from sediments under anoxic, but also oxic conditions and can date back to the Holocene and Pleistocene (Agnelli et al. 2007; Lejzerowicz et al. 2013). Such data can be useful to give insights into the evolutionary history of the studied species but have also been used to track human activities along the shores of an alpine lake (Giguet-Covex et al. 2014).

### exDNA as a target for biofilm treatment

Representing an attractive target for biofilm control, exDNA has been extensively studied and reviewed (e.g. Okshevsky and Meyer 2015; Okshevsky et al. 2015; Penesyan et al. 2015; Wnorowska et al. 2015). Next to its digestion with DNase (Aung et al. 2017; Bhongir et al. 2017; Brown et al. 2015a; Brown et al. 2015b; Rajendran et al. 2014; Waryah et al. 2017; Ye et al. 2017), also the use of antibodies to target the DNA-binding proteins (DNABII) located at the vertex of crossed exDNA strands was proposed (Brockson et al. 2014; Novotny et al. 2016; Rocco et al. 2017) in order to damage structural integrity and consequently increase susceptibility of the biofilm constituents to antibiotic agents. Similarly, several genes associated with the release of exDNA or with autolysis as well as quorum sensing inhibitors can be the target of an anti-biofilm therapy (e.g. Bao et al. 2015; Beltrame et al. 2015; Si et al. 2015; reviewed by Wolska et al. 2016). A nanomaterial cleaving exDNA of *S. aureus* biofilms was also proposed as a promising therapeutic material against biofilms (Thiyagarajan et al. 2016).

Deduced from its role as a main constituent of the EPS in biofilms, exDNA has been identified as a key contributor to uranium biomineralisation. It has been stated that the use of microorganisms producing exDNA in their biofilm may provide a cheap alternative to standard physiochemical treatment processes during the remediation of sites contaminated with radionuclides (Hufton et al. 2016).

In medical sciences, exDNA provides a useful tool for diagnostics as well as therapy monitoring, as its concentrations correlate with a variety of pathologies including cancer (Laktionov et al. 2004) and autoimmune disorders (Raptis and Menard 1980; reviewed by O’Driscoll 2007). Some studies also highlight the possibility to use DNase I to treat tumour cells as it targets the exDNA that facilitates the aggregation of the cells (Alekseeva et al. 2017; Hawes et al. 2015). During pregnancy, the entire foetal genome circulates in the maternal blood, enabling the non-invasive detection of foetal genetic disorders (Fan et al. 2012).

Interestingly, exDNA has also been found to be useful in forensics: using chemical force microscopy, exDNA can be located and quantified on the surface of human epithelial cells or on other surfaces, after a transfer through contact with skin and saliva. In that way, it provides a new tool in the forensic analysis of touch samples (Wang et al. 2017).

Concluding, it can be stated that exDNA was often attributed to mainly derive from dead cells; it has been shown that actively released exDNA makes up a quantitatively relevant fraction of the total exDNA pool of different environments. An active release also goes hand in hand with a better protection of the exDNA against DNases through the binding on different extracellular compartments such as minerals, lipids and proteins or through methylation (Böckelmann et al. 2006). Once arranged to the desired structure, such extracellular exDNA-containing complexes can perform a number of tasks in different environments, owed either to the sticky character of the electrically charged exDNA molecule, or to the information that the exDNA can bear for other cells (Fig. 1). Next to these functions, exDNA can also serve as a source of energy and nutrients to other cells after a fragmentation by DNases. All these properties of exDNA provide a great variety of possible applications that have been developed or are being developed across different fields of research.
